# Pulsed Estrogen Therapy Prevents Post-OVX Porcine Dura Mater Microvascular Network Weakening via a PDGF-BB-Dependent Mechanism

**DOI:** 10.1371/journal.pone.0082900

**Published:** 2013-12-09

**Authors:** Olga V. Glinskii, Virginia H. Huxley, Vladimir V. Glinskii, Leona J. Rubin, Vladislav V. Glinsky

**Affiliations:** 1 Research Service, Harry S. Truman Memorial Veterans Hospital, Columbia, Missouri, United States of America; 2 Department of Medical Pharmacology and Physiology, University of Missouri, Columbia, Missouri, United States of America; 3 Department of Pathology and Anatomical Sciences, University of Missouri, Columbia, Missouri, United States of America; 4 Department of Biomedical Sciences, University of Missouri, Columbia, Missouri, United States of America; 5 Center for Gender Physiology and Environmental Adaptation, University of Missouri, Columbia, Missouri, United States of America; 6 Yale Medical School, New Haven, Connecticut, United States of America; University of Pittsburgh School of Medicine, United States of America

## Abstract

In postmenopausal women, estrogen (E2) deficiencies are frequently associated with higher risk of intracranial hemorrhage, increased incidence of stroke, cerebral aneurysm, and decline in cognitive abilities. In younger postpartum women and those using oral contraceptives, perturbations in E2 are associated with higher risk of cerebral venous thrombosis. A number of serious intracranial pathologic conditions linked to E2 deficiencies, such as dural sinus thrombosis, dural fistulae, non-parenchymal intracranial hemorrhages, migraines, and spontaneous cerebrospinal fluid leaks, involve the vessels not of the brain itself, but of the outer fibrous membrane of the brain, the dura mater (DM). The pathogenesis of these disorders remains mysterious and how estrogen regulates structural and functional integrity of DM vasculature is largely unknown. Here, we demonstrate that post ovariectomy (OVX) DM vascular remodeling is manifested by microvessel destabilization, capillary rarefaction, increased vascular permeability, and aberrant angio-architecture, and is the result of disrupted E2-regulated PDGF-BB signaling within dura microvasculature. These changes, associated with the reduction in systemic PDGF-BB levels, are not corrected by a flat-dose E2 hormone replacement therapy (HRT), but are largely prevented using HRT schedules mimicking physiological E2 fluctuations. We demonstrate that 1) E2 regulates PDGF-BB production by endothelial cells in a dose-dependent manner and 2) optimization of PDGF-BB levels and induction of robust PDGF-mediated endothelial cell-vascular pericyte interactions require high (estrous) E2 concentrations. We conclude that high (estrous) levels of E2 are important in controlling PDGF-mediated crosstalk between endothelial cells and pericytes, a fundamental mechanism governing microvessel stability and essential for preserving intracranial homeostasis.

## Introduction

Microvascular networks adjust continuously to ever changing functional needs of an organ in response to myriad physiological and pathological processes. Such adaptation is highly regulated and kept in balance by the preexisting vascular architecture, hemodynamic forces exerted by the flowing blood, metabolic status of the tissue, as well as numerous cytokines, growth factors, and hormones including estrogens.

Sex hormone deficiencies are often associated with increased risk of several pathological conditions in the brain system including stroke and cerebral or dura mater aneurysms both leading to a higher risk of intracranial hemorrhage, dural sinus/cerebral vein thrombosis (CVT), and spontaneous cerebrospinal fluid (CSF) leaks [1], [2]. These conditions frequently result in significant neurologic morbidity and reduced cognitive abilities [3]. Epidemiological data indicate that in women the risk of intracranial aneurysm development and rupture rises during and after menopause [4]. Spontaneous intracranial hypotension and spontaneous CSF leaks due to dura mater (DM) weakening affect women twice as often as men, and the onset of symptoms occurs typically in the fourth or fifth decade of life [1], [2], [5]. Abnormal connections between dural arteries and veins within the leaflets of cranial dura mater, dural arteriovenous fistulae (DAVF), are viewed commonly as acquired lesions diagnosed in women over the age of 40 [6]–[8] and account for approximately 10–15% of all intracranial vascular malformations [9]. As these changes occur with higher incidence when ovarian hormone levels fall, their incidence has been attributed to hormonal factors [10], [11]. According to the recent recommendations of the American Heart Association on the evaluation and management of CVT, the highest three conditions predisposing women for this cerebrovascular complication are: 1. Use of oral contraceptives (54.3% prevalence); 2. Pro-thrombotic conditions (34.1% prevalence); and 3. Pregnancy or puerperium (21% prevalence) [12]. These data underscore the impact of perturbations in sex hormones on CVT development and affecting cerebrovenous outflow, one of the key factors in maintaining normal brain function.

Importantly, approximately 70% of intracranial blood volume is located within the venous compartment, the vast majority of which is situated within dura mater tissue. However, the role of non-cerebral, intracranial dura mater vasculature, critically involved in the regulation of cerebral venous outflow, CSF absorption [13]–[15], and control of intracranial pressure [16]–[18], has been largely overlooked. The unique nature of the meningeal vasculature possessing physiologic arteriovenous shunts and ability of neomembrane formation contributes to the genesis of multiple intracranial vascular malformations [19], [20].

It appears that vascular abnormalities in the intracranial dura mater, including asymptomatic ones, are more common than appreciated previously. Moreover, epidemiologically these changes are clearly associated with ovarian hormone commotions. Indeed, the pathophysiology of conditions such as migraine has been long associated with impaired functionality of dura mater microvasculature (including altered microvessel permeability) as well as sex hormone imbalances. The increase in vascular permeability [21], [22] and vasodilation of meningeal arterioles [23] induced by mast cell mediators have been suggested as putative mechanisms underlying vascular headaches such as migraine [24]. Further, recent studies demonstrate that migraine-associated recruitment and maturation of dura mater mast cells, usually located perivascularly [25], also critically depend on ovarian hormone production [26]. On the other hand, previous reports indicated that mast cell interactions with dura microvasculature are intimately involved in migraine-associated stress induced neurovascular inflammation manifested by increased microvessel permeability [27]. Thus, alterations in dura mater microvessel functionality contribute both directly and indirectly (via overall dura mater weakening) to the pathogenesis of neurovascular inflammation and macrovascular abnormalities described above. However, the effects of estrogen on the structure and function of meningeal microvasculature are largely unknown. Recently we demonstrated that surgical cessation of ovarian hormone production by ovariectomy (OVX) results in significant remodeling within porcine meningeal vasculature [28]. This remodeling, characterized by vessel destabilization, capillary rarefaction, increase in average microvessel size, and increase in vascular permeability [28], is indicative of impaired vascular functionality. In the present study we examined how two different regimens of the estrogen (E2) hormone replacement therapy (HRT), one maintaining diestrous E2 levels and one mimicking physiological E2 fluctuations associated with menstrual cycle, modified the ability to preserve dura mater microvascular networks following OVX *in vivo* and investigated the associated molecular mechanisms underpinning estrogen-dependent control of vascular stability and remodeling *in vitro*.

## Methods

### Animals and Hormone Replacement Therapy

Animal experiments were approved by the University of Missouri Institutional Animal Care and Use Committee (Protocol # 3733). Mature 9–12 month-old female Yucatan miniature swine were used in this study. Bilateral OVX was performed under general anesthesia by a team of surgeons and HRT with 17β-estradiol (E2) using Climara estradiol transdermal system patches (Berlex, Montwille, NJ, USA) was carried out at the National Center for Gender Physiology Swine Hormone Core. The following four experimental groups were used in this study: 1. Control, intact female animals (IF; N = 9); 2. Ovariectomized animals with no hormone replacement (OVX; N = 6); 3. Ovariectomized animals with flat dose HRT (OVX+FD HRT) using one 12.5 cm^2^ Climara patch containing 3.8 mg estradiol USP and providing 0.05 mg of E2 per day for maintaining diestrous E2 plasma levels, which was initiated immediately after OVX (N = 4). All animals in OVX+FD HRT group continuously received this dose of E2 throughout duration of the experiments (2 months); 4. Ovariectomized animals with pulsed HRT (OVX+P HRT), in which in addition to a constant basal dose of 0.05 mg of E2 per day for two months as in OVX+FD HRT group, animals were pulsed twice for 3 days beginning days 19 and 40 post OVX, respectively, with additional Climara patches doubling the E2 dose to mimic menstrual cycle (N = 5).

In all animals, blood collection was performed weekly using vascular ports installed at the time of surgery. In OVX+P HRT group, additional blood samples were collected daily during each three-day pulse E2 treatment. Plasma and serum samples were prepared and stored at −80°C until use. Plasma E2 levels were determined using ^125^I-estradiol radioimmunoassay kit (Diagnostic Systems Laboratories, Inc., Webster, TX, USA) according to manufacturer's instructions.

### Imaging

Animals were sacrificed two months post OVX, blood samples acquired and dura mater harvested and processed as described previously [29], [30]. Alexa Fluor 488-conjugated soybean agglutinin (SBA) in physiological Kreb's solution (30 µg/ml final concentration) was used to visualize the microvasculature. Dura mater terminal microvascular networks were imaged first using a fluorescence video microscopy system [Laborlux™ 8 microscope (Leitz Wetzlar, Germany) equipped with 75-watt xenon lamp and QICAM high performance digital CCD camera (Quantitative Imaging Corporation, Burnaby, Canada)], and then with a confocal system [IX-70™ Olympus Microscope equipped with Solamere™ confocal unit with dual laser line capabilities (50 mW 488 nm & 514 nm, 10 mW 457 nm) and acoustic coupler].

### Computer-Assisted Morphometric Analysis

The collected images were analyzed using the following software: ImageJ version 1.33u (NIH, Bethesda, MD, USA), Adobe Photoshop 7 (Adobe Systems Inc., San Jose, CA, USA), and Fovea Pro Photoshop-compatible plug-ins (Reindeer Graphics, Inc., Asheville, NC, USA). Fovea Pro™ stereology tools were used to analyze microvessel volume and surface-to volume ratio.

The changes in microvessel permeability were analysed from fluorescent dye accumulation within 10 µm diameter circular regions of interest (ROI) located in a perivascular space immediately adjacent to the microvessels of ≤10 µm in diameter. A total of 90 measurements were performed for each experimental group (10 ROI/frame, 3 frames/animal, 3 animals/group). For these purposes, fluorescent photomicrographs, acquired using exactly the same camera settings (gain, offset, and exposure time) after 30-min perfusion of the microvasculature with 30 µg/ml solution of Alexa Fluor 488-conjugated SBA lectin in physiological Kreb's solution (PKS) followed by a 30-min wash with PKS alone, were analyzed off-line. For each frame, the dye accumulation indices were calculated as the mean fluorescence intensity of each ROI divided by the background (the mean fluorescent intensity of the same size ROI located over the darkest unstained area of the frame).

**Figure 1:**
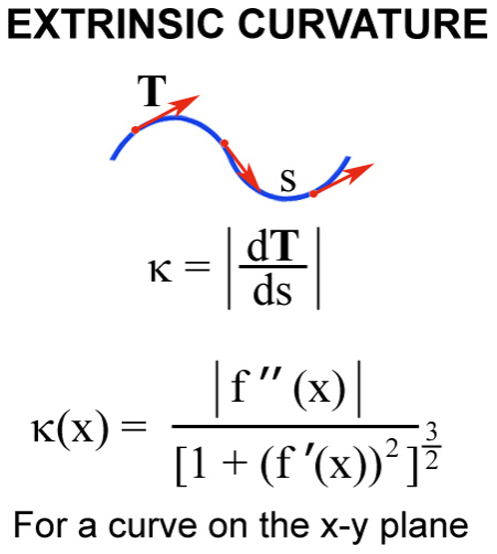
Vessel wall extrinsic curvature is used to describe nonlinearity of blood vessels. For the purpose of this study, extrinsic curvature of a line at a given point is a measure of how quickly the curve changes direction at that point and, therefore, is defined as the magnitude of the rate of change of the unit tangent vector T with respect to the arc length s. Thus, for the curve on the x-y plane, the extrinsic curvature k(x) is calculated by the formula shown.

In addition, a software tool was developed in house to aid with the analysis of morphological and architectural properties associated with changes in vessel wall curvature and microvessel nonlinearity. The program approaches mathematical analysis of capillary networks by interpolating points, generated along the blood vessel wall via user interface, with a system of cubic functions. The computational algorithm generates a system of cubic splines which, when graphed together, form continuous, differentiable function with a shape near identical to that of a blood vessel wall. With this model of the blood vessel wall in hand, the program computes the expression for the extrinsic curvature of the function at any point along the line and integrates the function from the initial to the terminal endpoint to yield the “total” microvessel curvature [31]. For the purpose of this study, the extrinsic curvature of a line (κ) at a given point (Fig. 1) is defined as a measure of how quickly the curve changes direction at that point i.e. as the magnitude of the rate of change of the unit tangent vector (T) with respect to the arc length (s). Thus, for the curve on the x-y plane, the extrinsic curvature κ(x) is defined by the formula provided in Fig. 1.

### Antibody Array

Human Growth Factor Antibody Array I (RayBiotech, Inc., Norcross, GA, USA) was used to analyze the relative levels of expression of 41 growth factors and receptors in the serum of the experimental animals according to manufacturer's instructions (see Tables S1 and S2 for the Antibody Array map and complete list of growth factors and receptors). Antibody arrays were probed with 1.0 ml of undiluted serum from IF, OVX, OVX+FD HRT, and OVX+P HRT swine. Chemiluminescent detection was performed on a ChemiDoc XRS system (Bio-Rad Laboratories, Hercules, CA, USA). The Discovery Series Quantity One 1-D Analysis Software and 12×8 array tool (Bio-Rad) were used for densitometry. Subsequent normalization of densitometry data was carried out using a RayBio® Antibody Array Analysis Tool (RayBiotech). Four serum samples from each experimental group were analyzed.

### Endothelial Cell (EC) culture

A microvascular brain endothelial cell line (bEnd.3) was purchased from ATCC (Manassas, VA). Cells were grown in Dulbecco's Modified Eagle's Medium (DMEM) supplemented with 10% (v/v) fetal bovine serum (FBS) and adjusted to contain 2 mM L-glutamine and 10 mM HEPES. Cells were maintained as a monolayer culture in a humidified incubator in 5% CO2/95% air at 37°C. For the estrogen-stimulation studies, cells were serum-deprived for 2 hours and treated with different doses of β-Estradiol (Sigma-Aldrich, St. Louise, MO) in phenol red-free DMEM supplemented with L-glutamine and 10 mM HEPES for 24 hours. Cells were lysed using CelLyticTMM Cell Lysis Reagent (Sigma-Aldrich) containing protease inhibitor cocktail (Sigma-Aldrich). The Quantikine PDGF-BB Immunoassay kit (R&D Systems, Minneapolis, MN) was used to measure PDGF-BB in the brain endothelial cell lysates according to manufacturer's instructions.

Human brain vascular pericytes (HBVP, ScienCell, Carlsbad, CA) were kindly provided by Dr. George Davis (University of Missouri School of Medicine). For 2-D tube assembly assay, bEnd.3 and HBVP cells were transfected to stably express green fluorescent protein (GFP) and red fluorescent protein (RFP) respectively using pre-made expression codon optimized lentiviral particles (GenTarget Inc., San Diego, CA) according to manufacturer's instruction. The resulting bEnd.3-GFP and HBVP-RFP cells were suspended in DMEM phenol-red free medium supplemented with L-glutamine, 10 mM HEPES at 5 and 1×10^4^ cell/ml respectively. Next, bEnd.3-GFP (30 µl) and HBVP-RFP (20 µl) cells were seeded into µ-Slide (ibidi LLC, Verona, WI) with Geltrex LDEV-Free reduced growth factor basement membrane matrix (Gibco Life Technologies). In addition, β-Estradiol (Sigma-Aldrich,) at 10^−11^ and 10^−10^ M concentrations, function blocking anti-PDGF-BB (Abcam, Cambridge, MA) and anti-VEGF (PeproTech, Rocky Hill, NJ) antibodies were added at 0.2 µg/ml and 0.1 µg/ml respectively. Mouse IgG1 (Santa Cruz Biotech) was used as an isotype specific control. After the cultures assembled for 24 hours, transmitted light and epifluorescence images were acquired using a 5× lens. Two independent experiments were performed in triplicates for each experimental condition.

### Statistical Analysis

The statistical analysis of data was performed using GraphPad Prism™ version 4 software (GraphPad Software, Inc., San Diego, CA, USA). One-way analysis of variance, Bartlett's test for equal variances, ANOVA table, Tukey's multiple comparison test, and Newman-Keuls multiple comparison test were used, as appropriate, to assess statistical significance of data. Bar graphs represent mean ± SD, or mean ± SEM as indicated in figure legends.

## Results

### Pulsed E2 treatment prevents post-OVX meningeal microvascular alterations

**Figure 2:**
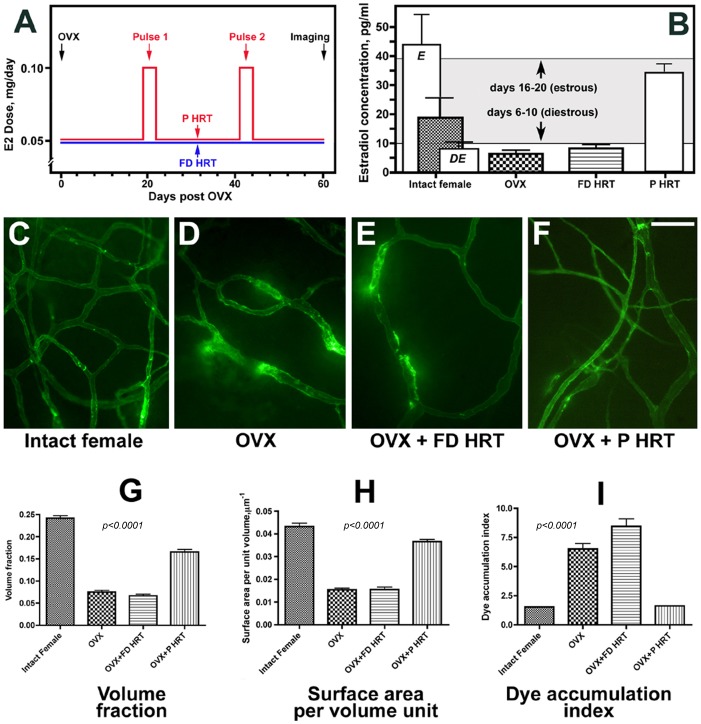
A, Timeline and E2 doses used in OVX+FD HRT (blue line) and OVX+P HRT (red line) experiments. B, Plasma levels of E2 in intact female, OVX, and OVX+HRT pigs. In intact female animals, in addition to the monthly average E2 level (filled bar), estrous and diestrous E2 levels are shown (open bars labeled E and DE respectively). The level of plasma E2 on the peak of a 3-day pulse is shown for OVX+P HRT group. Gray area indicates the magnitude of physiological fluctuations of plasma E2 levels as per literature. C through F, Dura mater microvascular networks of intact female Yucatan miniature pigs (C, N = 9), 2 months post OVX (D, N = 6), 2 months post OVX in animals receiving FD HRT with 0.05 mg of 17 β-estradiol per day supporting diestrous E2 levels (E, N = 4), and 2 months post OVX in animals receiving P HRT mimicking physiological fluctuations in E2 levels (F, N = 5). G through I, Response to OVX and different HRT regimens on a tissue volume occupied by terminal microvascular networks (G), blood vessel surface-to-volume ratio (H), and microvessel permeability (I). In G through I, bar graphs show mean ± standard error of mean. Scale bar shown in F, 100 µm. In B, G, H, and I, IF and OVX data adopted with permission from Ref. 28 to provide reference points for assessing the effects of FD HRT and P HRT.

In this study, we investigated whether HRT with 17 β-estradiol could prevent post OVX meningeal vascular remodelling manifested by capillary rarefaction, increased average microvessel size, and increased vascular permeability [28]. We have used two different HRT regimens to address this question: a flat-dose HRT (FD HRT) designed to maintain diestrous E2 levels, and a pulsed HRT (P HRT) mimicking physiological E2 fluctuations associated with a menstrual cycle (Fig. 2, A). In our experiments, estrous and diestrous E2 plasma levels in intact females were 43.8±6 and 8.1±1 pg/ml respectively (Fig. 2, B). Of note, E2 plasma levels in the post OVX animals showed only a marginal (1.2-fold) decrease from 8.1±1 pg/ml to 6.9±0.8 pg/ml compared with intact female diestrous levels, suggesting that diestrous E2 levels are largely maintained by extragonadal E2 production. FD HRT with 0.05 mg of 17 β-estradiol per day increased E2 levels in post OVX animals to 9.2±0.9 pg/ml. Although the fact that E2 plasma level in FD HRT group did not differ significantly from the OVX group could be viewed as a limitation of this study, our purpose for the FD HRT regimen was to maintain diestrous E2 plasma level. FD HRT increased uterus size compared to post-OVX females without HRT (data not shown), but failed to prevent dura mater vascular remodeling (Fig. 2, C through I). In the OVX+P HRT group, the E2 plasma level on a peak of a 3-day pulse was 32.9±3.6 pg/ml, and external sexual organs of these experimental animals showed changes associated with heat, further suggesting a successful mimicking of a menstrual cycle. Remarkably, in contrast to FD HRT, mimicking physiological E2 fluctuations with P HRT prevented almost completely post OVX remodeling of the meningeal microvascular networks (Fig. 2, C through I).

Computer-assisted morphometric analysis (Fig. 2, G through I) revealed a greater than 3-fold decrease (*p*<0.001) in a tissue volume occupied by terminal microvascular networks in OVX animals compared to the intact control females (Fig. 2, G). FD HRT failed to prevent post OVX microvessel loss (Fig. 2, G), whereas P HRT preserved microvessel density at 68.5% of the intact female level (Fig. 2, G). A significant (almost 3-fold) decrease in the calculated blood vessel surface-to-volume ratio (Fig. 2, H) in both OVX and OVX+FD HRT swine (*p*<0.001) indicates that the observed overall decrease in terminal dura mater microvessel density was due to a prevalent loss of capillaries. This change was also accompanied by an apparent increase in the average microvessel size. As with the volume fraction, FD HRT did not prevent changes in this parameter either. In contrast, P HRT preserved surface area per unit volume at 84.5% of the level of intact animals (Fig. 2, H). Finally, with respect to microvessel function, there was a greater than 4-fold increase in perivascular fluorescent dye accumulation in OVX pigs compared to intact females (Fig. 2, I), indicative of changes in vascular permeability. This change in barrier function was even further aggravated by FD HRT (*p*<0.001 between intact female and OVX and OVX+FD HRT groups; *p*<0.01 between OVX and OVX+FD HRT groups). Remarkably, barrier function was preserved by P HRT, which completely prevented changes in microvascular permeability (Fig. 2, I).

### Changes in vessel architecture and morphology suggest molecular mechanisms involved in post OVX remodeling of DM microvasculature

**Figure 3:**
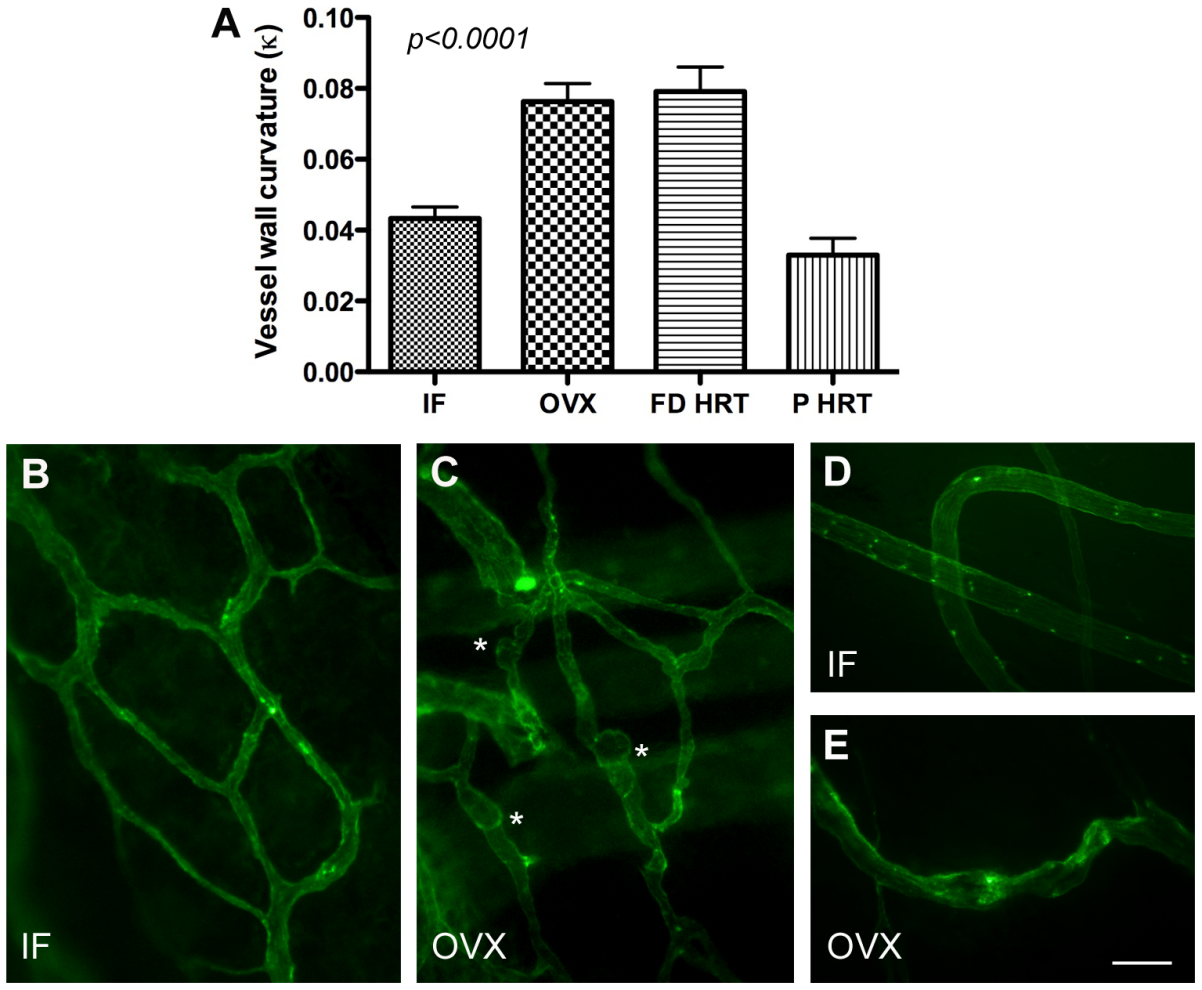
A, changes in microvessel wall intrinsic curvature in response to OVX, FD HRT, and P HRT. Data presented as mean ± standard error of mean. B and C, Formation of multiple focal dilations/microaneurysms (asterisks) in DM microvasculature following OVX (C) compared to intact female (IF) animals (B). D and E, Reduced pericyte decoration of pig meningeal microvessels following OVX (E) compared with intact females (IF) animals (D) as revealed by fluorescent microscopy using mesenchymal progenitor marker SBA (Soybean Agglutinin).

Quantitative analysis of blood vessel wall nonlinearity revealed significant architectural and morphological changes in the microvessel wall following OVX (Fig. 3). Our data show that cessation of ovarian hormone production in OVX females leads to a significant increase in extrinsic curvature (Fig. 3, A) of microvessel walls (*p*<0.0001) with formation of focal dilations/microaneurysms (Fig. 3, B and C) and dramatically reduced pericyte decoration (Fig. 3, D and E). These architectural and morphological changes (increased blood vessel nonlinearity, microaneurysm formation, and lack of pericyte decoration) show striking similarity with the vascular morphology of PDGF-B-deficient mouse embryos [32] prompting us to hypothesize that estrogen control of DM vascular remodeling involves the regulation of PDGF-mediated signaling. Indeed, PDGF-BB mediated signaling is critical for pericyte and undifferentiated mural cell precursor recruitment [33], which are integral to microvessel maturation and stabilization [33]–[35]. Hence, mice deficient in PDGF-B or PDGFRβ display aberrant vessel architecture and an absence of pericytes in the microvasculature [34].

### Pulsed E2 treatment prevents an imbalance in systemic PDGF-AB and -BB levels in post OVX animals

**Figure 4:**
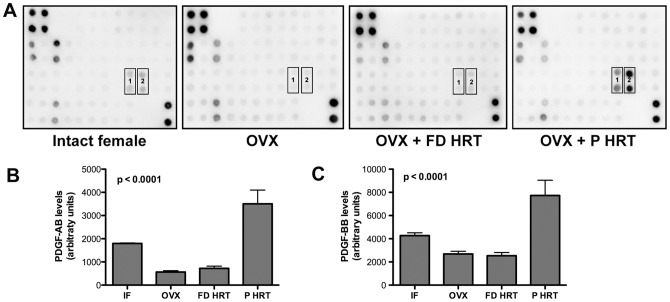
Changes in PDGF-AB and -BB serum levels following OVX and different HRT regimens. Representative images of antibody array analysis of 41 growth factors and receptors following OVX and different HRT regimens in serum of intact female, OVX, OVX+FD HRT, and OVX+P HRT animals (4 samples were analyzed for each group) are shown in A. Two months post OVX, levels of PDGF-AB and -BB (boxes 1 and 2 on antibody arrays respectively) consistently exhibit dynamics correlated with changes in the status of meningeal microvessels (decreased in OVX animals compared to intact females, not corrected by FD HRT, and normalized by P HRT).

Using antibody array, we analyzed 41 growth factors and receptors including PDGF-AA, -AB, and –BB on systemic (serum) level. In these analyses, the most pronounced changes were consistently registered in expression levels of PDGF-AB and -BB (Fig. 4) without significant changes in PDGF-AA expression (data not shown). Changes in PDGF-AB (Figure 4, A box 1) and PDGF-BB (Figure 4, A box 2) expression levels were consistent with the status of meningeal microvascular networks and were decreased in OVX animals compared to intact females, not corrected by FD HRT, and largely normalized by P HRT (Figure 4, B for PDGF-AB and C for PDGF-BB). These data demonstrate that without estrous E2 spikes, PDGF-BB levels decrease significantly and could be not sufficient to support endothelial/pericyte interactions essential for maintaining microvascular adaptation and stability.

### Estrogen stimulates endothelial PDGF-BB production in a dose-dependent manner

**Figure 5:**
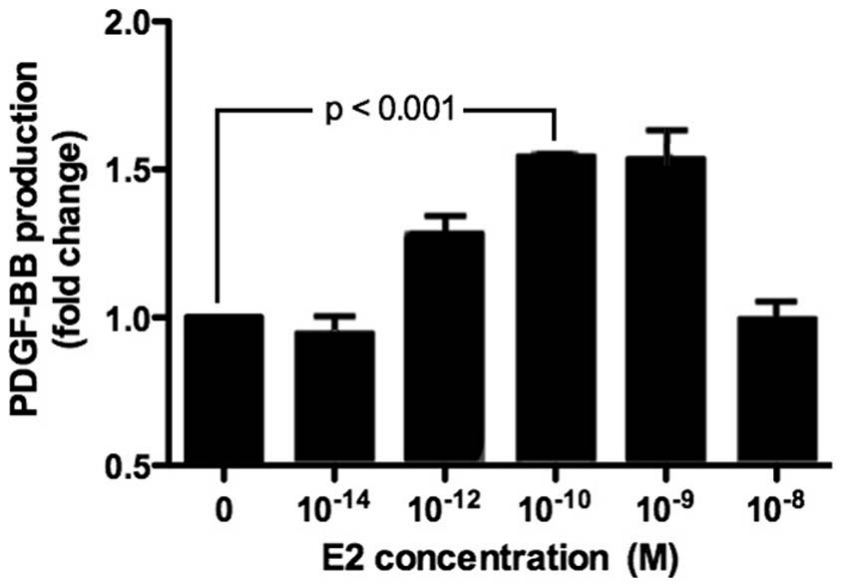
Dose-dependent changes in PDGF-BB production by brain endothelial cells in response to E2 treatment. Brain endothelial cells bEnd.3 were treated with indicated E2 concentrations for 24-BB ELISA.

Within the vascular tissue, PDGF-BB is produced by endothelial cells controlling a paracrine regulation of PDGF-mediated crosstalk between endothelial cells and pericytes. PDGF-BB governs pericyte recruitment to and retention at the microvascular networks in a process viewed as the fundamental mechanism of microvessel maturation and stabilization [35], [36]. Thus we investigated next whether PDGF-BB production by endothelial cells depends on E2 levels. The *in vitro* experiments employing bEnd.3 brain microvascular EC demonstrated that 24 hour treatment with increasing E2 concentrations resulted in dose-dependent changes in PDGF-BB production by brain EC (Fig. 5). Importantly, the PDGF-BB expression was maximal at E2 concentrations corresponding to estrous E2 levels *in vivo*. However, PDGF-BB expression returned to basal level when E2 was elevated beyond physiological concentrations (Fig. 5). These data underscore the importance of estrous E2 levels in maximizing PDGF-B synthesis by endothelial cells and support our hypothesis that a decrease in systemic levels of PDGF-AB and BB detected *in vivo* in post OVX animals (Fig. 4) is, in part, a consequence of the E2-dependent decline in PDGF-B production by endothelial cells.

### High (estrous) E2 levels mediate EC/pericyte interactions in PDGF-BB dependent manner

**Figure 6:**
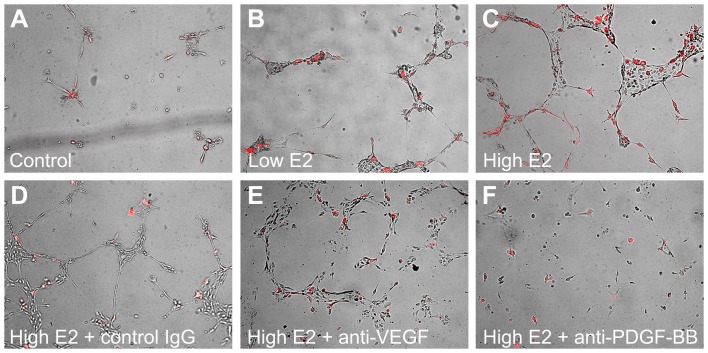
Brain endothelial cells bEnd.3 and brain vascular pericytes HBVP (red) do not form 3-D networks under basal conditions (A). Low diestrous E2 level (10^−11^ M) only marginally improves pericyte association with endothelial cells (B). In the presence of high estrous E2 level (10^−10^ M) bEnd.3 and HBVP form vast 3-D networks within 24 h (C). Neither control IgG (D) nor function blocking anti-VEGF antibody (E) inhibit microtubular network formation by brain endothelial cells and brain vascular pericytes in response to high E2 concentration. Function blocking anti-PDGF-BB antibody completely abolishes the ability of brain endothelial cells and brain vascular pericytes to form microtubular networks in response to high E2 level (F).

To investigate further whether estrogen controls EC/pericyte interactions via PDGF-mediated mechanisms, we performed 2-D co-culture experiments using bEnd.3 brain EC and HBVP brain pericytes (Fig. 6). Remarkably, while control cultures (Fig. 6, A) did not form EC/pericyte microtubular networks, and cell treatment with low (diestrous) E2 levels only marginally improved EC/pericyte association (Fig. 6, B), bEnd.3 and HBVP cells formed vast microtubular networks in the presence of the high (estrous) E2 concentration (Fig. 6, C) without any additional stimulation with angiogenic/growth factors. The ability of brain EC and pericytes to form microtubular networks in response to stimulation with estrous E2 levels was not affected by application of the control IgG or function-blocking antibody to VEGF (Fig. 6, D and E). However, introduction of the anti-PDGF-BB function-blocking antibody (Fig. 6, F) completely abolished tube formation. These results unambiguously demonstrate that E2 controls microtubular network formation by EC and pericytes in a PDGF-BB-dependent manner and that estrous E2 concentrations are required to mediate PDGF-BB production sufficient to induce EC/pericyte interaction.

## Discussion

As demonstrated herein, estrogen-dependent microvascular changes associated with the loss of ovarian hormone secretion may constitute a morphological basis for the development of multiple perimenopausal symptoms and provide a mechanism whereby postmenopausal women become more susceptible to cardiovascular and cerebrovascular dysfunction. Our data show that remodeling in the meningeal microvasculature associated with cessation of the ovarian hormone cycling may have a profound effect on intracranial vessel function. Rarefaction of the microcirculation (a loss of capillary area) is one form of vascular remodeling that promotes hypertension and tissue ischemia [37]. Capillary rarefaction of this magnitude inevitably results in corresponding increases in peripheral vascular resistance [38], requires microvascular network accommodation to altered hemodynamic and water-solute exchange requirements and affects vascular function. Indeed, capillary rarefaction in dermal and skeletal muscle microvasculature, leading to the increase in peripheral resistance and altered vascular function, is characteristic of hypertensive animals [39] and humans [38].

Increased leakage from capillaries in OVX animals is likely detrimental to dura mater tissue for several reasons: (i) Accumulation of interstitial fluid increases the diffusion distance for oxygen and nutrients as well as limits the diffusional removal of potentially toxic byproducts of cellular metabolism leading to local tissue hypoxia and cytotoxicity; (ii) Within the intracranial space, which is limited by surrounded cranial bones, relatively small increments in transcapillary fluid filtration induce large increases in interstitial fluid pressure. This, in turn, reduces the vascular transmural pressure gradient and physically compresses capillaries, thereby reducing nutritive tissue perfusion [40] leading to further tissue hypoxia, a state detrimental to tissue function potentially resulting in DM tissue weakening and tearing with subsequent localized CSF leaks leading to spontaneous intracranial hypotension.

In addition to capillary rarefaction and increased microvascular leakage, our data demonstrate that loss of estrogen leads to microvascular changes in dura mater similar to those observed in diabetic microangiopathy, such as the formation of microaneurysms (Fig. 3). These changes are associated with a reduction in systemic PDGF-BB levels, which most likely compromise perivascular cell PDGF-BB/PDGFRβ signaling. Several studies show that, whereas the complete knockouts of PDGF-B or PDGFRβ are lethal during late embryonic development or at birth [41], [42], the exchange of the intracellular domain of PDGFRβ for that of PDGFRα produces viable mice but with a severe deficit in retinal pericytes and who subsequently develop retinopathy [43]. It has been suggested that pericyte loss may cause local mural weakening leading to outpouchings (microaneurysms) in the capillary wall [44]. Indeed, mice deficient in PDGF-B or PDGFRβ display aberrant vessel remodeling, an absence of pericytes in the microvasculature, and development of numerous microaneurysms with rupturing at late gestation [32], [34], [41], [42]. Other studies on the cardiovascular defects in PDGF-B and PDGFRβ mutant mice have also demonstrated the critical importance of PDGF-B/PDGFRβ signaling for proper pericyte recruitment in developmental angiogenesis in a number of different organs, including the retina [45]–[49].

Within the vascular tissue, PDGF-BB is produced by EC, while PDGRβ is expressed in pericytes, enabling for a paracrine regulation of PDGF-mediated crosstalk between the two cell types governing pericyte recruitment to and retention at the microvascular networks in a process viewed as the fundamental mechanism of microvessel maturation and stabilization [35], [36]. The *in vitro* experiments employing bEnd.3 (brain microvascular EC) demonstrated that treatment with increasing E2 concentrations resulted in dose-dependent changes in PDGF-BB production by brain EC (Fig. 5). Importantly, this effect increased with elevating doses and was maximal at E2 concentrations corresponding to estrous E2 levels *in vivo*. Furthermore, treatment of brain EC/brain pericyte co-cultures with estrous (but not diestrous) E2 concentrations was sufficient to induce robust EC/pericyte interactions and microtubular network formation without stimulation with any exogenous growth factors (Fig. 6), and this process was clearly PDGF-BB-dependent. There are several important implications of these results. Specifically, they suggest that 1) Decreases in the systemic levels of PDGF-AB and BB detected *in vivo* in post OVX animals (Fig. 4) are a consequence of the E2-dependent decline in PDGF production by endothelial cells; and 2) High (estrous) physiological E2 levels are essential for inducing PDGF-mediated crosstalk between EC and vascular pericytes facilitating pericyte recruitment and microvessel stabilization. Thus, alterations in E2 control of PDGF-mediated crosstalk between EC and pericytes could be responsible for network destabilization and loss of cerebral microvessels associated with the onset of reproductive senescence in women. They also provide a mechanistic basis for understanding a differential role for high (estrous) and low (diestrous) levels of E2 in controlling PDGF-mediated crosstalk between brain endothelial cells and pericytes, a fundamental mechanism governing microvessel maturation and stabilization.

Estrogen-dependent remodeling of meningeal microvasculature is an extremely complex process tightly regulated on multiple levels. Our previous results demonstrated that estrogen receptor alpha (ER-α) dependent decline in angiopoietin-1 expression could be responsible in part for a post OVX destabilization of dural microvessels and increase in their permeability [28]. Recent reports from other groups indicate that dura mater mast cells could contribute to the increase in meningeal microvessel permeability [24], and this could be also estrogen-dependent [26]. In addition to a described herein E2-dependent control of PDGF-B production by endothelial cells controlling EC/pericyte interactions, PDGF-B expression could be also regulated by a hypoxia [50]. Indeed, we have reported recently that following the initial estrogen-dependent loss of meningeal microvessels in post OVX animals, hypoxia-driven stromal responses upregulate local PDGF-B production via HIF-1α controlled mechanism and induce PDGF/VEGF system activation promoting angiogenic activity [51]. Whether and to what extent such responses could compensate for a decrease in estrogen-controlled PDGF production due to perturbations in sex hormone levels in postmenopausal and post OVX women remains to be elucidated.

In premenopausal women, estrogen creates in a timely manner two very distinct microenvironments: a high concentration of short duration and a low concentration for a much longer time corresponding to the menstrual cycle. It appears that regular physiological oscillations in E2 levels associated with the estrous cycle are vitally important for programming target cells and systems for complex medium- and long-term tasks associated with vascular maintenance and angio-adaptation hence preservation of healthy microvascular networks. Elucidating the details of such programming and accounting for the complex integrative nature of estrogen effects is essential for avoiding undesirable consequences of HRT [52]. Revising HRT regimens toward those resembling the natural E2 dynamics may offer new solutions for controlling and preventing cardiovascular and cerebrovascular diseases.

## Supporting Information

Table S1 Human Growth Factor Antibody Array I Map.
(DOC)

Table S2 Abbreviations Used in Human Growth Factor Antibody Array I Map.
(DOC)
