# Supplementary material for: Pulsed Estrogen Therapy Prevents Post-OVX Porcine Dura Mater Microvascular Network Weakening via a PDGF-BB-Dependent Mechanism
Source: PLoS One. 2013 Dec 9;8(12):e82900. doi: 10.1371/journal.pone.0082900 (PMC3857298; doi:10.1371/journal.pone.0082900)
Supplement: Table S1 — Human Growth Factor Antibody Array I Map. (DOC) [file pone.0082900.s001.doc]

**Table S1.** Human Growth Factor Antibody Array I Map

|  | **A** | **B** | **C** | **D** | **E** | **F** | **G** | **H** | **I** | **J** | **K** | **L** |
| --- | --- | --- | --- | --- | --- | --- | --- | --- | --- | --- | --- | --- |
| **1** | POS | POS | NEG | NEG | AR | bFGF | b-NGF | EGF | EGF R | FGF-4 | FGF-6 | FGF-7 |
| **2** | POS | POS | NEG | NEG | AR | bFGF | b-NGF | EGF | EGF R | FGF-4 | FGF-6 | FGF-7 |
| **3** | GCSF | GDNF | GM-CSF | HB-EGF | HGF | IGFBP-1 | IGFBP-2 | IGFBP-3 | IGFBP-4 | IGFBP-6 | IGF-I | IGF-I SR |
| **4** | GCSF | GDNF | GM-CSF | HB-EGF | HGF | IGFBP-1 | IGFBP-2 | IGFBP-3 | IGFBP-4 | IGFBP-6 | IGF-I | IGF-I SR |
| **5** | IGF-II | M-CSF | M-CSF R | NT-3 | NT-4 | PDGF Rα | PDGF Rβ | PDGF-AA | PDGF-AB | PDGF-BB | PlGF | SCF |
| **6** | IGF-II | M-CSF | M-CSF R | NT-3 | NT-4 | PDGF Rα | PDGF Rβ | PDGF-AA | PDGF-AB | PDGF-BB | PlGF | SCF |
| **7** | SCF R | TGF-α | TGF-β | TGF-β2 | TGF-β3 | VEGF | VEGF R2 | VEGF R3 | VEGF-D | BLANK | BLANK | POS |
| **8** | SCF R | TGF-α | TGF-β | TGF-β2 | TGF-β3 | VEGF | VEGF R2 | VEGF R3 | VEGF-D | BLANK | BLANK | POS |
