# Supplementary material for: Pulsed Estrogen Therapy Prevents Post-OVX Porcine Dura Mater Microvascular Network Weakening via a PDGF-BB-Dependent Mechanism
Source: PLoS One. 2013 Dec 9;8(12):e82900. doi: 10.1371/journal.pone.0082900 (PMC3857298; doi:10.1371/journal.pone.0082900)
Supplement: Table S2 — Abbreviations Used in Human Growth Factor Antibody Array I Map. (DOC) [file pone.0082900.s002.doc]

**Table S2.** Abbreviations Used in Human Growth Factor Antibody Array I Map

| **Abbreviation** | **Factor** |  | **Abbreviation** | **Factor** |
| --- | --- | --- | --- | --- |
| **POS** | Positive Control |  | **IGF-II** | Insulin Like Growth Factor 2 |
| **NEG** | Negative Control |  | **M-CSF** | Macrophage Colony Stimulating Factor |
| **AR** | Amphiregulin |  | **M-CSF R** | Macrophage Colony Stimulating Factor Receptor |
| **bFGF** | Basic Fibroblast Growth Factor |  | **NT-3** | Neurotrophin-3/Nerve Growth Factor-2 |
| **b-NGF** | Nerve Growth Factor Beta |  | **NT-4** | Neurotrophin-4 |
| **EGF** | Epidermal Growth Factor |  | **PDGF Rα** | Platelet Derived Growth Factor Receptor Alpha |
| **EGF R** | Epidermal Growth Factor Receptor |  | **PDGF Rβ** | Platelet Derived Growth Factor Receptor Beta |
| **FGF-4** | Fibroblast Growth Factor 4 |  | **PDGF-AA** | Platelet Derived Growth Factor AA |
| **FGF-6** | Fibroblast Growth Factor 6 |  | **PDGF-AB** | Platelet Derived Growth Factor AB |
| **FGF-7** | Fibroblast Growth Factor 7 |  | **PDGF-BB** | Platelet Derived Growth Factor BB |
| **GCSF** | Granulocyte Colony Stimulating Factor |  | **PlGF** | Placental Growth Factor |
| **GDNF** | Glial Cell Line-Derived Neurotrophic Factor |  | **SCF** | Stem Cell Factor |
| **GM-CSF** | Granulocyte-Macrophage Colony Stimulating Factor |  | **SCF R** | Stem Cell Factor Soluble Receptor |
| **HB-EGF** | Heparin-Binding EGF-Like Factor |  | **TGF-α** | Transforming Growth Factor Alpha |
| **HGF** | Hepatocyte Growth Factor |  | **TGF-β** | Transforming Growth Factor Beta |
| **IGFBP-1** | Insulin Like Growth Factor Binding Protein 1 |  | **TGF-β2** | Transforming Growth Factor Beta 2 |
| **IGFBP-2** | Insulin Like Growth Factor Binding Protein 2 |  | **TGF-β3** | Transforming Growth Factor Beta 3 |
| **IGFBP-3** | Insulin Like Growth Factor Binding Protein 3 |  | **VEGF** | Vascular Endothelial Growth Factor |
| **IGFBP-4** | Insulin Like Growth Factor Binding Protein 4 |  | **VEGF R2** | Vascular Endothelial Growth Factor Receptor 2/flk-1 |
| **IGFBP-6** | Insulin Like Growth Factor Binding Protein 6 |  | **VEGF R3** | Vascular Endothelial Growth Factor Receptor 3/flt-4 |
| **IGF-I** | Insulin Like Growth Factor 1 |  | **VEGF-D** | Vascular Endothelial Growth Factor D |
| **IGF-I SR** | Insulin Like Growth Factor 1 Soluble Receptor |  | **BLANK** | BLANK |
